# Supplementary material for: Neuregulin 4 Downregulation Alters Mitochondrial Morphology and Induces Oxidative Stress in 3T3-L1 Adipocytes
Source: Int J Mol Sci. 2024 Oct 31;25(21):11718. doi: 10.3390/ijms252111718 (PMC11546241; doi:10.3390/ijms252111718)
Supplement: Supplementary file 1 [file ijms-25-11718-s001.zip › ijms-3258461-Supplementary.pdf]

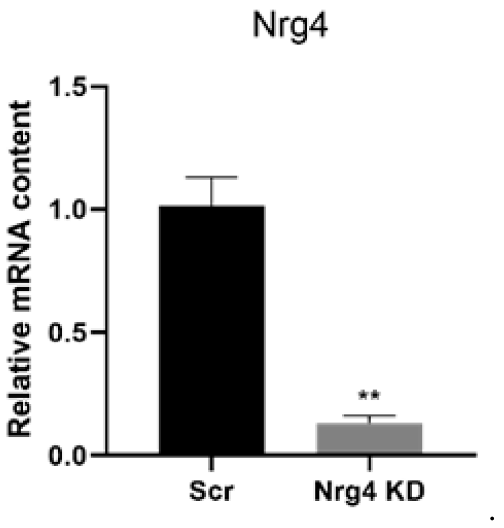

Figure S1. Expression of *Nrg4* in scrambled and Nrg4 KD adipocytes.

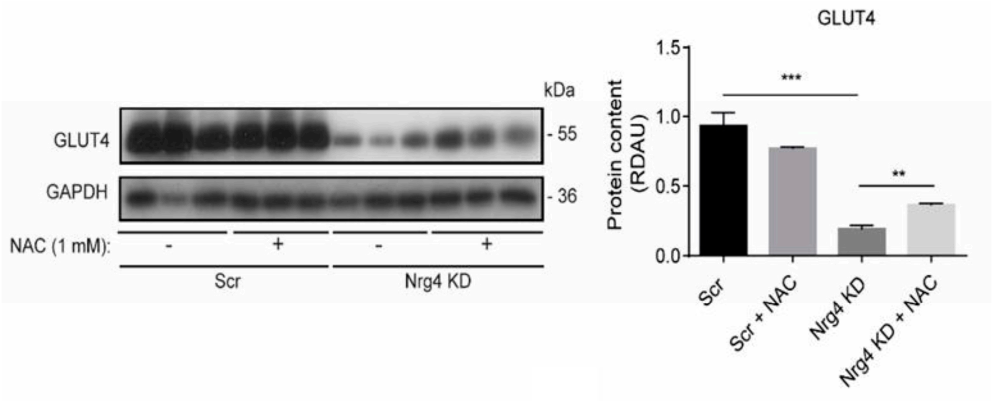

Figure S2. Effect of the antioxidant N-acetylcysteine on GLUT4 glucose transporters' protein content in scrambled and Nrg4 KD adipocytes. Table S1: Primer sequences used for quantitative-PCR analysis.
